# Exploring attitudes towards more sustainable dentistry among adults living in the UK

**DOI:** 10.1038/s41415-022-4910-6

**Published:** 2022-08-26

**Authors:** Harriet M. Baird, Steven Mulligan, Thomas L. Webb, Sarah R. Baker, Nicolas Martin

**Affiliations:** 4141510346001 Department of Psychology, University of Sheffield, UK; 4141510346002 School of Clinical Dentistry, University of Sheffield, UK

## Abstract

**Introduction ** Despite evidence that public pressure can promote sustainability in various domains (for example, retail and travel), no research has considered the public's attitudes towards sustainability in dentistry.

**Methods ** A questionnaire was developed to measure attitudes towards sustainable dentistry among adults living in the UK and their willingness to make compromises to reduce the impact of their dental treatment on the environment. In total, 344 adults completed the questionnaire that also measured pro-environmental identity and concern, general willingness to make compromises for the environment, and the tendency to engage in ecological behaviours.

**Results ** Participants reported positive attitudes towards sustainable dentistry, and were willing to compromise their time, convenience and durability of their dental treatment, as well as pay more, to reduce the impact of their dental work on the environment. Participants were not willing to compromise their health or the aesthetics of their teeth. There was also evidence that participants' current oral health shaped their attitudes towards sustainable dentistry, such that better oral health was associated with more positive attitudes towards more sustainable dentistry.

**Conclusions** Given that public pressure can be a significant driver of change, these findings provide valuable insight into the kind of compromises that may be accepted by the public in order to improve the sustainability of dental services.

## Key points

- This is the first study to consider the public's attitudes towards sustainability in dentistry and their willingness to make compromises in order to reduce the impact of their dental treatments on the environment.
- The findings suggest that people have relatively positive attitudes towards sustainability in dentistry and are willing to make some compromises in order to reduce the impact of their dental treatments on the environment (eg time, convenience, financial) but not others (eg health and aesthetics).
- The findings also suggest that people's current oral health shapes their attitudes towards sustainable dentistry and their willingness to make compromises, such that better oral health was associated with more positive attitudes towards more sustainable dentistry.

## Introduction

Dentistry is a resource-intensive field that has a significant impact on the environment.^1^ Each year, dental clinics generate significant quantities of plastic waste,^2^ use gallons of water^3^ and use substantial amounts of electricity.^4^ Furthermore, in 2013 to 2014, dental services in England operated by the National Health Service (NHS) produced approximately 675 kilotonnes of greenhouse gas emissions.^5^ As a result of the Climate Change Act (2008), the NHS is legally required to reduce its greenhouse gas emissions by 80% by 2050 (a target that has recently been revised to net zero),^6^ and sustainability is now an important consideration for all healthcare services.

To achieve significant and long-term improvements in the sustainability of dental services, engagement with multiple stakeholders is needed to drive change and reduce the risk of unintended consequences.^7^^,^^8^ For example, if dental services are to significantly reduce their carbon emissions, then policy and legislation are required to set goals and enforce standards (for example, an 80% reduction by 2050),^6^ manufacturing and supply of dental products and devices must be carried out as sustainably as possible (using data and environmental analysis to guide decision-making),^9^ and patients and healthcare professionals must be educated in sustainable choices (for example, encourage active travel or use of public transport).^10^

However, previous research exploring sustainability in dentistry has focused on the role of healthcare providers,^11^ and no research to date has considered the role of the public in meeting these challenges. This omission is important because research in other contexts has demonstrated that public pressure can be a significant driver of change.^12^^,^^13^ Thus, evaluating the public's attitudes towards sustainability in dentistry may help to understand where changes to practice would be accepted and supported by the public, as well as identifying the potential for public pressure to prompt governments, companies and organisations into action.

However, acting more sustainably often requires that people compromise their time or money, and/or exert more effort to obtain the desired outcome. Furthermore, while research has suggested that people tend to view pro-environmental goals as important, research has also shown that people consider their health as more important.^14^^,^^15^ A key question, therefore, is whether people are still willing to make compromises for the environment when the trade-offs involve factors related to their health and/or the benefits of their dental treatment (for example, the costs, quality, or aesthetics of their treatment).

## The present research

The aim of the present research was to explore attitudes towards sustainable dentistry among adults living in the UK and their willingness to make compromises in order to reduce the impact of their dental treatments on the environment. A number of different trade-offs were considered relating to time and convenience, money, functionality, aesthetics and health. These trade-offs were hypothetical in nature such that they aimed to assess what compromises people would be willing to make if given the option. For example, if an environmental assessment revealed that having a longer appointment could reduce the number of single-use plastics used (for example, by allowing the use of reusable instruments that could be sterilised between uses), then would patients be willing to compromise their time to reduce the waste generated from their treatment? Similarly, some of the questions asked participants if they would be willing to compromise their health in order to achieve more sustainable dentistry. Although in practice, a patient's health would never be compromised (and indeed, we did not expect participants to indicate that they would be willing to make this compromise), measuring a range of different trade-offs allowed us to compare people's willingness to make different compromises and empirically test the hypothesis that people prioritise their health.^14^^,^^15^

The research also sought to identify factors that were associated with participants' attitudes and willingness to make compromises for more sustainable dentistry (for example, sample demographics, use of dental services, individual differences in pro-environmental attitudes). Thus, there were two overarching research questions for the present research:

1. To what extent do people have positive attitudes towards more sustainable dentistry and to what extent are they willing to make compromises to achieve it?
2. What factors are associated with peoples' attitudes towards, and willingness to make compromises for, more sustainable dentistry?

## Materials and methods

### Study design

An online questionnaire, distributed via the survey software Qualtrics (https://www.qualtrics.com/), was used to measure participants' attitudes towards more sustainable dentistry and their willingness to make compromises to reduce the impact of their dentistry on the environment, along with potentially related constructs - such as pro-environmental identity and concern, willingness to make sacrifices for the environment more generally, and the tendency to engage in pro-environmental behaviour in other domains. There were two versions of the questionnaire: i) a short version consisting of only the measures of demographics and attitudes towards, and willingness to make compromises for, sustainable dentistry; and ii) a full version comprising all measures. Participants recruited in private dental practices were asked to complete the short version of the questionnaire so that they had time to complete the questionnaire before or shortly after their appointment. Participants recruited via other means completed the full questionnaire. Data were collected over six months between August 2020 and February 2021, and ethical approval for the study was obtained from the Research Ethics Committee in the Department of Psychology at The University of Sheffield (March 2020, #033450).

### Recruitment and participant characteristics

Three strategies were used to recruit participants: i) staff and dentists at four private dental practices in the UK asked patients who were attending their dental appointment whether they would be interested in taking part in a research study and (if so) sent them a link to the online questionnaire; ii) an advert was placed on Prolific (https://www.prolific.co/), an online participant recruitment platform for scientific research; and iii) adverts were placed on the social media accounts (for example, Facebook and Twitter) of members of the research team. Participants recruited via Prolific received £2.50 for completion of the questionnaire, whereas participants recruited in dental practices or via social media had the option to enter a prize draw to win a £20 Amazon voucher. To be eligible to participate, individuals needed to be aged 18 or over and be currently living in the UK.

Table 1 displays the demographic and dental characteristics of the 344 participants who were recruited. Participants were aged between 18 and 77 years (*M* = 33.0; *SD* = 12.1) and were predominantly female (70%) and White British (84%). Most participants described themselves as registered with a dental practice (82%), had visited a dentist within the last 12 months (69%) and attended the dentist regularly for routine check-ups (59%). On average, participants rated their oral health as good (*M* = 3.7 out of 5; *SD* = 0.8) and the participants who said that they were currently experiencing pain with their teeth (11%) rated that pain as mild (*M* = 3.8 out of 10; *SD* = 2.1).

**Table 1:** Characteristics of the sample

Characteristics	N (missing)	%	Mean (SD)
**Gender**	**344 (0)**		
Male	105	30.5	
Female	239	69.6	
**Age (years)**	**343 (1)**		**33.0 (12.1)**
**Ethnicity**	**343 (1)**		
White British	290	84.3	
Other	53	15.4	
Country of birth	344 (0)		
UK	291	84.6	
Other	53	15.4	
**Level of education**	**343 (1)**		
No formal education	3	0.9	
Primary education	2	0.6	
Secondary education	31	9.0	
College/sixth form	114	33.1	
Undergraduate degree	120	34.9	
Postgraduate degree	54	15.7	
PhD/Doctorate	19	5.5	
**Employment status**	**344 (0)**		
Full-time	144	41.9	
Part-time	74	21.5	
Unemployed	22	6.4	
Student	75	21.8	
Retired	10	2.9	
Unable to work	3	0.9	
Other (for example, at home caring for family)	16	4.7	
**Recruitment method**	**344 (0)**		
Prolific	304	88.4	
Social media	13	3.8	
Dental practice	27	7.8	
**Currently registered with a dentist**	**344 (0)**		
Yes	281	81.7	
No	63	18.3	
**Last visit to the dentist**	**344 (0)**		
Within the last month	37	10.8	
Within the last 6 months	65	18.9	
Within the last 12 months	136	39.5	
Within the last 3 years	63	18.3	
3 years or more	41	11.9	
Never been to the dentist	2	0.6	
**Frequency of dental visits**	**344 (0)**		
Never been to the dentist	2	0.6	
When I have a dental problem	138	40.1	
Regularly for check-ups	204	59.3	
**Payment for dental treatment**	**343 (1)**		
NHS	214	62.2	
Private (for example, dental insurance)	106	30.8	
I don't pay for my dental treatments	13	3.8	
I don't go to the dentist	10	2.9	
**Are teeth currently causing pain?**	**344 (0)**		
Yes	36	10.5	
No	308	89.5	
If yes, how bad is the pain on a scale of 1-10?	34 (2)		3.82 (2.10)
**How would you rate your oral health (on a scale from 1-5)?**	**344 (0)**		**3.68 (0.82)**
Some of the groups were combined for the purpose of the analyses to reduce small and unequal group sizes. For the level of education, the lowest three levels (i.e., 'no formal education', 'primary education', and 'secondary education') were combined, as were the upper two levels (i.e., 'postgraduate degree' and 'PhD/ doctorate'). Within employment status, the groups 'unemployed', 'retired' and 'unable to work' were combined, and the group 'other', was excluded.			

### Measures and procedure

Participants were invited to take part in a study exploring people's views towards sustainable dentistry. If participants were interested in taking part, then they were asked to click on a link to the online questionnaire, which first presented information about the study, followed by a consent form. Participants were then asked to provide demographic information, including their age, gender and ethnicity, along with questions regarding how frequently they visited the dentist, when their last visit to the dentist was and how they paid for their dental treatment. Participants were also asked to indicate whether their teeth were currently causing them pain (and if so, how they would rate the pain on a ten-point scale, where ten is the worst pain imaginable) and how they would rate their general oral health (on a five-point scale ranging from 'very poor' to 'very good'). Following this, participants were asked to complete measures of pro-environmental attitudes and behaviours. Table 2 provides an overview of the measures used in the present research. The measures were presented in a random order and, unless stated otherwise, all items were assessed on five-point Likert scales ranging from 'strongly disagree' to 'strongly agree'. A list of the questionnaire items that have not been used in previous research (that is, items to assess participants' attitudes towards sustainable dentistry, and willingness to make compromises to reduce the impact of their dental treatment on the environment) are provided in Appendix 1.

**Table 2:** Summary of measures used in the present study

**Construct/measure**	**Items**	**Example items**	**α**
Attitudes toward sustainable dentistry The extent to which people have positive attitudes towards more sustainable dentistry	8	'It is important to me that my dental treatments do not harm the environment' and 'It is good to visit a dentist that cares about the environment'	0.91
Willingness to compromise time and convenience for more sustainable dentistry The extent to which people would compromise their time and convenience to reduce the impact of their dental treatments on the environment	7	'I would be willing to wait longer for an appointment if my dental treatments were better for the environment' and 'I would be willing to visit a more environmentally friendly dentist even if it was more inconvenient to me'	0.85
Willingness to pay more for more sustainable dentistry The extent to which people would pay more to reduce the impact of their dental treatments on the environment	3	'I would be willing to pay extra to reduce the impact of my dental treatments on the environment' and 'I would be willing to pay more if my dental treatments were better for the environment'	0.77
Willingness to compromise the durability of one's dental treatment for more sustainable dentistry The extent to which people would compromise the durability of their dental treatments to reduce the impact on the environment	3	'I would be willing to have a filling that didn't last as long if it was better for the environment' and 'I would be willing to have a filling repaired rather than replaced, if it was better for the environment'	0.83
Willingness to compromise the appearance of one's teeth for more sustainable dentistry The extent to which people would compromise the appearance of their teeth to reduce the impact on the environment	5	'I would be willing to have more noticeable dental work if it was better for the environment' and 'I would be willing to have a less than perfect smile if it was better for the environment'	0.81
Willingness to compromise oral health for more sustainable dentistry The extent to which people would compromise their oral health to reduce the impact of their dental treatments on the environment	2	'I would be willing to compromise the health of my teeth for the environment' and 'I would not be willing to compromise the health of my teeth for the environment (reverse scored)'	0.58
Pro-environmental identity^16^ The extent to which people believe that acting in a pro-environmental way is part of their self-identity	4	'I think of myself as someone who is very concerned with environmental issues' and 'I think of myself as an environmentally friendly consumer'	0.68
Pro-environmental concern^17^ The extent to which people have a general concern for the environment	4	'Compared to other things in my life, environmental problems are not that important to me' and 'Environmental problems are of great concern to me personally'	0.77
Ecological worldview^18^^,^^19^ The degree to which participants endorse an ecological worldview (that is, the belief that human beings are part of nature rather than separate from it)	15	'We are approaching the limit of the number of people the earth can support' and 'Humans are severely abusing the environment'	0.83
Willingness to sacrifice for the environment scale^20^ The extent to which participants would sacrifice more immediate self-interests, exert more effort, or accept costs for the benefit of the environment. Three further items were taken from the World Values Survey^21^ to assess participants' willingness to make financial sacrifices for the environment	8	'I am willing to give things up that I like doing if they harm the natural environment', 'Even when it is inconvenient to me, I am willing to do what I think is best for the environment' and 'I would give part of my income if I were certain that the money would be used to prevent environmental pollution'	0.86
Pro-environmental behaviour^22^ The extent to which participants engage in pro-environmental behaviours in other domains (for example, saving energy at home)	26	'Do you reuse your shopping bags?', 'Do you eat meat?' and 'Are you a member of an environmental organisation?'	0.81
Note: Attitudes towards more sustainable dentistry and willingness to make compromises were measured by items developed by the study authors (who comprise a multidisciplinary team of clinicians and researchers in dentistry and psychology) through discussion and reviews of the literature.^23^ A full list of these items can be found in Appendix 1. All items were measured on 5-point Likert scales ranging from 'strongly disagree' to 'strongly agree', with the exception of the measure of pro-environmental behaviour which was measured on a 3-point Likert scale, with the labels 'never', 'sometimes' and 'always'. Furthermore, as some of the items assessing pro-environmental behaviour were not applicable to all participants (for example, 'Do you buy organic meat products?' is only applicable to people who eat meat), the label 'not applicable' was also added to the response options. We then calculated each participant's average based on the number of items that they answered (that is, excluding 'not applicable' responses), such that higher scores indicated more pro-environmental behaviour. A full description of all of the measures used in the present research can be found online (https://tinyurl.com/attdentistry).			

### Approach to analyses

The data were analysed in two stages. First, descriptive statistics were used to explore participants' attitudes towards sustainable dentistry and their willingness to make compromises to achieve it. Second, the relationships between the demographic variables (for example, age, gender), use of dental services (for example, frequency of dental visits), and individual differences (for example, pro-environmental identity and concern) and participants' attitudes and willingness to make compromises for more sustainable dentistry were explored using correlations, t-tests, and (M)ANOVAs as appropriate. The analyses were conducted using SPSS (version 25), and the anonymised data and syntax relating to the analyses can be found online (https://tinyurl.com/attdentistry) The full statistics (for example, *p* values, effect sizes) from all analyses can be found in Appendix 2.

## Results

### To what extent do participants have positive attitudes towards sustainable dentistry and are willing to make compromises to achieve it?

Table 3 presents the descriptive statistics for each study variable. Overall, participants reported relatively positive attitudes towards sustainability in dentistry (*M* = 3.8, 95% CI [3.7, 3.9]) and were moderately willing to compromise time and convenience (*M* = 3.7, 95% CI [3.6, 3.8]), the durability of their dental treatments (*M* = 3.2, 95% CI [3.1, 3.3]) and to pay more (*M* = 3.1, 95% CI [3.1, 3.3]) for more sustainable dentistry. However, it was less clear whether participants were willing to compromise the appearance of their teeth (*M* = 2.8, 95% CI [2.7, 2.9] or their oral health (*M* = 2.2, 95% CI [2.2, 2.3]).

**Table 3:** Descriptive statistics for study variables

**Variables**	**N**	**Mean**	**95% CI**	**Min**	**Max**
Attitudes towards sustainable dentistry	344	3.8	3.7, 3.9	1.0	5.0
Willingness to compromise time and convenience	341	3.7	3.6, 3.8	1.0	5.0
Willingness to pay more	343	3.1	3.0, 3.3	1.0	5.0
Willingness to compromise the durability of dental treatments	343	3.2	3.1, 3.3	1.0	5.0
Willingness to compromise appearance of teeth	343	2.8	2.7, 2.9	1.0	5.0
Willingness to compromise health	343	2.2	2.2, 2.3	1.0	5.0
Pro-environmental identity	317	4.0	3.9, 4.0	1.5	5.0
Pro-environmental concern	317	3.7	3.6, 3.8	1.0	5.0
Ecological worldview (NEP)	317	3.8	3.8, 3.9	2.3	5.0
Willingness to make sacrifices for the environment	316	3.6	3.5, 3.7	1.0	4.9
Pro-environmental behaviour	317	2.2	2.2, 2.2	1.4	2.9
Key: N = sample size; CI = confidence interval; NEP = New Ecological Paradigm					

There was a significant difference in the extent to which participants were willing to make different compromises for more sustainable dentistry, *F*(3.43, 1165.68) = 240.14, *p* <0.001, *η*_p_^2^ = 0.41. Participants were significantly more willing to compromise their time and convenience than they were to pay more or compromise the durability of their dental treatments. Participants were least willing to accept compromises related to the appearance or health of their teeth. All comparisons were significant at *p* <0.001, except for the comparison between participants' willingness to pay more and their willingness to compromise the durability of their dental treatments (*p* = 1.00).

### Factors associated with participants' attitudes towards, and willingness to make compromises for, sustainable dentistry

#### Demographics

Participants' ethnicity, level of education and employment status were not found to be associated with their attitudes towards, or willingness to make compromises for, sustainable dentistry (*p* values >0.05). There were, however, significant differences according to age and gender, such that older participants were less willing to compromise their time and convenience than younger participants (*r* = -0.12, *p* = 0.029), and women had more positive attitudes towards sustainable dentistry (*M* = 3.9, 95% CI [3.8, 3.9]) compared to men (*M* = 3.6, 95% CI [3.5, 3.8]; *t*(169.75) = -2.08, *p* = 0.039, *d* = 0.26). Men and women did not differ in their willingness to make compromises for more sustainable dentistry (*p* values >0.05).

Given that our sample was recruited using three different methods (that is, Prolific, social media, or approached in a dental clinic), we explored whether participants' attitudes and willingness to make compromises varied according to how participants were recruited. There were significant differences between the methods of recruitment in participants' attitudes towards sustainable dentistry (*F*[2,338] = 6.78, *p* = 0.001, *η*_p_^2^ = 0.04) and willingness to pay more for more sustainable dentistry (*F*[2,338] = 10.76, *p* <0.001, *η*_p_^2^ = 0.06). Pairwise comparisons (with Bonferroni adjustment) revealed that participants who were recruited via dental practices had more positive attitudes towards sustainable dentistry (*M* = 4.3, 95% CI [4.0, 4.5]) than participants who were recruited via Prolific (*M* = 3.7, 95% CI [3.6, 3.8]; *p* = 0.006). Participants who were recruited via dental practices were also more willing to pay more to reduce the impact of their dentistry on the environment (*M* = 3.8; 95% CI [3.5, 4.1]) than participants who were recruited from Prolific (*M* = 3.1; 95% CI [3.0, 3.2]; *p* = 0.001).

#### Use of dental services

Participants who were registered with a dentist had more positive attitudes towards sustainable dentistry (*M* = 3.8, 95% CI [3.7, 3.9]) than participants who were not registered with a dentist (*M* = 3.6, 95% CI [3.4, 3.8]; *t*(342) = 2.00, *p* = 0.046, *d* = -0.28). Registration status was not associated with the extent to which participants were willing to make compromises to reduce the impact of their dentistry on the environment (*p* values >0.05). However, the frequency with which participants visited the dentist was found to be associated with the extent to which participants were willing to pay more to reduce the impact of their dentistry on the environment (*t*[339] = -3.04, *p* = 0.003, *d* = 0.34). Participants who visited the dentist regularly for routine check-ups were more willing to pay more to reduce the impact of their dentistry (*M* = 3.3, 95% CI [3.2, 3.4]) than participants who only visited the dentist when they had a dental problem (*M* = 3.0, 95% CI [2.8, 3.1]). Given that only two participants reported that they never went to the dentist (see Table 2), this group was omitted from the analyses. The frequency with which participants visited the dentist was not associated with their attitudes towards sustainable dentistry, nor their willingness to compromise their time and convenience nor the health and aesthetics of their teeth (*p* values >0.05). There was no difference in participants' attitudes nor willingness to make compromises for more sustainable dentistry according to whether participants receive their dental treatments via the NHS or through a private provider (*p* values >0.05).

Correlation analyses indicated that participants' self-rated oral health was positively associated with their attitudes towards sustainable dentistry, such that better oral health was associated with more positive attitudes (*r* = 0.11, *p* = 0.042). Participants' oral health was not associated with their willingness to make compromises for more sustainable dentistry (*p* values >0.05). There was no difference in participants' attitudes towards sustainable dentistry nor their willingness to make compromises to reduce the impact of their dentistry according to whether or not participants were experiencing pain or discomfort with their teeth (*p* values >0.05)

#### Individual differences

Table 4 presents the bivariate correlations between the study variables. The correlations were all significant, except for the correlation between participants' ecological worldview (that is, scores on the NEP) and their willingness to compromise the health of their teeth for more sustainable dentistry (*r* = -0.03, *p* = 0.571). Attitudes towards sustainable dentistry were positively associated with participants' willingness to compromise their time and convenience (*r* = 0.68), money (*r* = 0.53) and the durability of their dental treatments (*r* = 0.52). Attitudes towards sustainable dentistry were also positively associated with participants' willingness to compromise the aesthetics (*r* = 0.40) and health of their teeth (*r* = 0.29), although the size of these relationships were smaller. Together, these findings suggest that (as expected) more positive attitudes are associated with greater willingness to make compromises to reduce the impact of dentistry on the environment.

**Table 4:** Correlations between study variables (all correlations were significant except those indicated within the key)

**Variables**	**1**	**2**	**3**	**4**	**5**	**6**	**7**	**8**	**9**	**10**	**11**
1. Attitudes towards sustainable dentistry	1.00	0.68	0.53	0.52	0.40	0.29	0.64	0.68	0.40	0.67	0.56
2. Willingness to compromise time and convenience		1.00	0.56	0.64	0.48	0.34	0.54	0.57	0.39	0.69	0.48
3. Willingness to pay more			1.00	0.55	0.40	0.30	0.39	0.45	0.28	0.64	0.44
4. Willingness to compromise the durability of dental treatments				1.00	0.53	0.36	0.44	0.45	0.27	0.56	0.39
5. Willingness to compromise appearance of teeth					1.00	0.54	0.30	0.32	0.13^*^	0.49	0.42
6. Willingness to compromise health						1.00	0.14^*^	0.25	-0.03^**^	0.32	0.26
7. Pro-environmental identity							1.00	0.74	0.52	0.65	0.57
8. Pro-environmental concern								1.00	0.53	0.63	0.58
9. Ecological worldview (NEP)									1.00	0.41	0.38
10. Willingness to make sacrifices for the environment										1.00	0.59
11. Pro-environmental behaviour											1.00
Key: * = p <0.05 (correlation not significant) ** = p = 0.571 (correlation not significant) NEP = New Ecological Paradigm											

Attitudes towards more sustainable dentistry were also positively associated with measures of pro-environmental identity and concern (*r *= 0.64 and 0.68, respectively). Similarly, willingness to make sacrifices for the environment more generally was also associated with participants' willingness to make compromises for more sustainable dentistry, although the correlation with willingness to compromise health (*r* = 0.49) was smaller than the correlations with other trade-offs (*r* values range from 0.56 to 0.69). Lastly, whether participants engaged in pro-environmental behaviour in other domains (for example, when buying groceries) was also positively associated with participants' attitudes towards sustainable dentistry and participants' willingness to make compromises for more sustainable dentistry (*r* values range from 0.26 to 0.56).

## Discussion

The aim of the present research was to explore attitudes towards sustainable dentistry among adults living in the UK and their willingness to make compromises in order to reduce the impact of their dental treatments on the environment. Participants in the present study reported positive attitudes towards more sustainable dentistry and were willing to compromise their time, convenience and the durability of their dental treatment in order to reduce the impact of their dental treatments on the environment. They were also willing to pay more if it meant that their dentistry was more sustainable. However, participants were less willing to compromise the appearance or health of their teeth, which is consistent with previous research indicating that, while people consider environmental goals important, they consider their health as more important.^14^^,^^15^

We also found that more positive attitudes towards sustainable dentistry were associated with greater willingness to make compromises in order to reduce the impact of dental treatments on the environment, with the size of these correlations ranging from medium to large. This is consistent with previous research and models of behaviour (for example, Theory of Planned Behaviour,^24^ Prototype Willingness Model)^25^ which suggest that people's attitudes are a key (albeit indirect) predictor of subsequent behaviour and suggest that interventions which focus on increasing positive attitudes may subsequently prompt people to be more willing to make greater compromises for the environment. In particular, research has shown that promoting specific attitudes (for example, towards sustainable dentistry) is the strongest predictor of subsequent behaviour (termed the principle of compatibility),^26^ so interventions might want to focus on promoting positive attitudes towards sustainable dentistry, rather than sustainability more generally.

The present study also explored factors that may be associated with (and thus help to understand) participants' attitudes towards, and willingness to make compromises for, more sustainable dentistry. Of the sample demographics, age and gender were found to be associated with participants' attitudes and willingness to make compromises. Specifically, younger participants were more willing to compromise their time and convenience in order to reduce the impact of their dental treatment on the environment, and women had more positive attitudes towards more sustainable dentistry. These findings are consistent with previous research that found that younger generations are more pro-environmentally driven^27^ and research that has pointed towards an 'eco gender gap', where men report being less committed to maintaining an eco-friendly lifestyle than women.^28^

In terms of participants' use of dental services, we found that participants who were registered with a dentist had more positive attitudes towards sustainable dentistry than participants who were not registered with a dentist, and participants who visited the dentist regularly for routine check-ups were more willing to pay more to reduce the impact of their dental treatments than participants who only visited the dentist when they had a dental problem. Similarly, participants who were recruited in a dental clinic had more positive attitudes and were more willing to pay more to reduce the impact of their dental treatments on the environment than participants who were recruited online. There are a number of possible explanations for these findings. It could be that people who are more engaged with dental services (that is, are registered with a dentist and visit the dentist more frequently) are more aware of the impact of dentistry on the environment (for example, because they have recently seen the high quantities of single-use plastics used during their appointment). However, it would be interesting to explore whether - and to what extent - people are aware of the materials that are used in their treatment.

Alternatively, it could be that people who are registered with a dentist and attend regularly for check-ups have better oral health and are therefore more willing to engage in sustainability efforts because doing so is likely to be less costly for them personally. In support of this explanation, we also found that better oral health was associated with more positive attitudes towards sustainable dentistry, and further exploratory analyses of our data confirmed that people who were registered with a dentist and attended the dentist more regularly also had better oral health. Taken together, these findings may suggest that if people have good (oral) health, then they are more willing to engage in sustainability efforts. However, if people have poor (oral) health, then they prioritise their health and are less willing to engage in sustainability efforts. Although these hypotheses would need to be empirically tested, they would support other arguments that good oral health can act as a driver towards more sustainable dentistry. For example, better oral health would lead to fewer appointments, fewer patient journeys, and less need for materials and intervention.^29^

Finally, whether participants engaged in pro-environmental behaviour in other domains (for example, recycling at home) was also positively associated with participants' attitudes towards sustainable dentistry and their willingness to make compromises for more sustainable dentistry. These findings support psychological theories which suggest that people need consistency in their attitudes, beliefs and behaviours (for example, Theory of Cognitive Dissonance)^30^ and supports research which has shown that pro-environmental behaviours tend to 'spill over' into other pro-environmental behaviours (for example, buying organic produce in the supermarket can lead to using less water at home).^31^

### Limitations and future directions

This is the first study to assess the public's attitudes towards sustainability in dentistry in the UK and provides useful insights into the types of compromises that people may be willing to make in order to reduce the impact of their dental treatments on the environment. However, the study is not without some limitations. For example, although we sampled the views of around 350 individuals, the composition of the sample may limit the generalisability of the findings. For example, the majority of the sample were female, White British and currently living in the UK. We only recruited adults who were currently living in the UK because previous research has indicated that there are cross-cultural differences in people's pro-environmental attitudes and behaviours,^32^ and the aim of the present research was to examine the attitudes and opinions of adults in the UK. However, the methods and tools used in the present research could be used to investigate and compare attitudes towards sustainable dentistry in other contexts and cultures. It is also worth noting that the present study did not measure household income nor socioeconomic status. These variables are potentially important, particularly as we are asking people whether or not they may be willing to make financial compromises to reduce the impact of their dental treatments on the environment.

The present study also assessed participants' willingness to make compromises, as opposed to actual behaviour; in part, because there is currently little choice for the public when it comes to reducing the impact of their dental treatments on the environment. However, understanding willingness is important as it can point to what people are likely to do if given the option - indeed, research has shown that willingness is a key predictor of behaviour in the future.^33^ Furthermore, given that the trade-offs that were examined in the present research were largely hypothetical in nature, future research may want to use environmental assessment (for example, Life Cycle Assessment),^34^ in order to inform which types of compromises would have a beneficial impact on the sustainability of dental services. Such research would inform what changes should be made, while our research can inform whether such changes would likely be accepted by the public.

Finally, the timing of the data collection for this study also situates the findings in a unique context. The data were collected in August 2020, approximately five months after the UK initiated a national lockdown in response to the COVID-19 pandemic. During this time, public health was prioritised, along with the economic and social impacts of COVID-19, and concerns regarding the environment were largely sidelined.^35^ As a result, the UK saw a significant increase in single-use plastics in both healthcare and beyond (for example, face masks and shields in retail)^36^ and many efforts to promote sustainability in other domains were abandoned (for example, Starbucks refused reusable coffee cups).^37^ In this context, it is promising that participants' attitudes towards improving the sustainability of dental services was largely positive in the present study; however, future research may want to consider whether and how participants' attitudes towards sustainable dentistry change over time.

## Conclusion

This is the first study to assess the public's attitudes towards more sustainable dentistry in the UK, and assess the extent to which people are willing to make personal compromises in order to reduce the impact of their dental treatments on the environment. Overall, we found that people have positive attitudes towards more sustainable dentistry and that people are willing to make certain compromises (for example, time, convenience and paying more), but not others (for example, health and aesthetics). The findings also suggest that people's current health may play an important role in their attitudes towards sustainable dentistry and their willingness to make compromises, such that only people who are in good health are willing to engage in sustainability efforts.

**Table:** ***Appendix 2*** Factors associated with participants' attitudes towards, and willingness to make compromises for, sustainable dentistry

**Factor**		**Statistical test**	**Result**
**Demographics**			
**Age**	Attitudes towards sustainable dentistry	Pearson's correlation	*r*(343) = 0.08, *p* = 0.159
	Willingness to compromise time and convenience		*r*(340) = -0.12, *p* = 0.029^*^
	Willingness to pay more		*r*(342) = 0.06, *p* = 0.235
	Willingness to compromise the durability of dental treatments		*r*(342) = -0.03, *p* = 0.640
	Willingness to compromise appearance of teeth		*r*(342) = 0.08, *p* = 0.168
	Willingness to compromise health		*r*(342) = 0.03, *p* = 0.577
**Gender**	Attitudes towards sustainable dentistry	Independent t-test	*t*(169.75) = -2.08, *p* = 0.039, *d* = 0.26^*^
	Willingness to compromise time and convenience		*t*(160.05) = -1.68, *p* = 0.094, *d* = 0.22
	Willingness to pay more		*t*(341) = -1.70, *p* = 0.091, *d* = 0.20
	Willingness to compromise the durability of dental treatments		*t*(341) = -0.34, *p* = 0.737, *d* = 0.04
	Willingness to compromise appearance of teeth		*t*(341) = 0.98, *p* = 0.328, *d* = -0.12
	Willingness to compromise health		*t*(341) = -0.09, *p* = 0.929, *d* = 0.01
**Ethnicity**	Attitudes towards sustainable dentistry	Independent t-test	*t*(341) = -0.58, *p* = 0.560, *d* = 0.09
	Willingness to compromise time and convenience		*t*(338) = 0.15, *p* = 0.884, *d* = -0.02
	Willingness to pay more		*t*(340) = 0.88, *p* = 0.378, *d* = -0.13
	Willingness to compromise the durability of dental treatments		*t*(340) = 0.19, *p* = 0.847, *d* = -0.03
	Willingness to compromise appearance of teeth		*t*(340) = -0.43, *p* = 0.665, *d* = 0.07
	Willingness to compromise health		*t*(340) = -0.75, *p* = 0.456, *d* = 0.11
**Level of education**	Attitudes towards sustainable dentistry	Between-subjects ANOVA	*F*(3,336) = 1.33, *p* = 0.264, *η*_*p*_^2^ = 0.01
	Willingness to compromise time and convenience		*F*(3,336) = 2.58, *p* = 0.053, *η*_*p*_^2^ = 0.02
	Willingness to pay more		*F*(3,336) = 1.69, *p* = 0.169, *η*_*p*_^2^ = 0.02
	Willingness to compromise the durability of dental treatments		*F*(3,336) = 2.16, *p* = 0.093, *η*_*p*_^2^ = 0.02
	Willingness to compromise appearance of teeth		*F*(3,336) = 1.34, *p* = 0.260, *η*_*p*_^2^ = 0.01
	Willingness to compromise health		*F*(3,336) = 1.27, *p* = 0.285, *η*_*p*_^2^ = 0.01
**Employment status**	Attitudes towards sustainable dentistry	Between-subjects ANOVA	*F*(3,321) = 0.31, *p* = 0.822, *η*_*p*_^2^ = 0.00
	Willingness to compromise time and convenience		*F*(3,321) = 1.53, *p* = 0.206, *η*_*p*_^2^ = 0.01
	Willingness to pay more		*F*(3,321) = 1.52, *p* = 0.209, *η*_*p*_^2^ = 0.01
	Willingness to compromise the durability of dental treatments		*F*(3,321) = 0.70, *p* = 0.556, *η*_*p*_^2^ = 0.01
	Willingness to compromise appearance of teeth		*F*(3,321) = 0.67, *p* = 0.573, *η*_*p*_^2^ = 0.01
	Willingness to compromise health		*F*(3,321) = 0.55, *p* = 0.650, *η*_*p*_^2^ = 0.01
**Recruitment method**	Attitudes towards sustainable dentistry	Between-subjects ANOVA	*F*(2,338) = 6.78, *p* = 0.001, *η*_*p*_^2^ = 0.04^*^
	Willingness to compromise time and convenience		*F*(2,338) = 1.31, *p* = 0.271, *η*_*p*_^2^ = 0.01
	Willingness to pay more		*F*(2,338) = 10.76, *p* <0.001, *η*_*p*_^2^ = 0.06^*^
	Willingness to compromise the durability of dental treatments		*F*(2,338) = 1.86, *p* = 0.157, *η*_*p*_^2^ = 0.01
	Willingness to compromise appearance of teeth		*F*(2,338) = 0.45, *p* = 0.636, *η*_*p*_^2^ = 0.00
	Willingness to compromise health		*F*(2,338) = 1.75, *p* = 0.176, *η*_*p*_^2^ = 0.01
**Use of dental services**			
**Registered with a dentist**	Attitudes towards sustainable dentistry	Independent t-test	*t*(342) = 2.00, *p* = 0.046, *d* = -0.28^*^
	Willingness to compromise time and convenience		*t*(339) = -0.97, *p* = 0.332, *d* = 0.14
	Willingness to pay more		*t*(341) = 0.31, *p* = 0.759, *d* = -0.04
	Willingness to compromise the durability of dental treatments		*t*(341) = -0.15, *p* = 0.882, *d* = 0.02
	Willingness to compromise appearance of teeth		*t*(341) = -1.67, *p* = 0.096, *d* = 0.23
	Willingness to compromise health		*t*(341) = -0.13, *p* = 0.900, *d* = 0.02
**Frequency of dental visits**	Attitudes towards sustainable dentistry	Independent t-test	*t*(340) = -1.82, *p* = 0.070, *d* = 0.20
	Willingness to compromise time and convenience		*t*(337) = 1.03, *p* = 0.305, *d* = -0.11
	Willingness to pay more		*t*(339) = -3.04, *p* = 0.003, *d* = 0.34^*^
	Willingness to compromise the durability of dental treatments		*t*(339) = -0.88, *p* = 0.378, *d* = 0.10
	Willingness to compromise appearance of teeth		*t*(339) = 0.75, *p* = 0.453, *d* = -0.08
	Willingness to compromise health		*t*(339) = 1.82, *p* = 0.069, *d* = -0.20
**Payment**	Attitudes towards sustainable dentistry	Independent t-test	*t*(318) = -0.46, *p* = 0.643, *d* = 0.06
	Willingness to compromise time and convenience		*t*(315) = 0.39, *p* = 0.701, *d* = -0.05
	Willingness to pay more		*t*(317) = -1.58, *p* = 0.116, *d* = 0.19
	Willingness to compromise the durability of dental treatments		*t*(317) = -0.25, *p* = 0.801, *d* = 0.03
	Willingness to compromise appearance of teeth		*t*(317) = 0.67, *p* = 0.505, *d* = -0.08
	Willingness to compromise health		*t*(317) = 0.56, *p* = 0.579, *d* = -0.07
**Last visit to dentist**	Attitudes towards sustainable dentistry	Between-subjects ANOVA	*F*(4,334) = 2.74, *p* = 0.029, *ηp*^2^ = 0.03^*^
	Willingness to compromise time and convenience		*F*(4,334) = 2.35, *p* = 0.053, *ηp*^2^ = 0.03
	Willingness to pay more		*F*(4,334) = 4.24, *p* = 0.002, *ηp*^2^ = 0.05^*^
	Willingness to compromise the durability of dental treatments		*F*(4,334) = 1.53, *p* = 0.195, *ηp*^2^ = 0.02
	Willingness to compromise appearance of teeth		*F*(4,334) = 0.73, *p* = 0.573, *ηp*^2^ = 0.01
	Willingness to compromise health		*F*(4,334) = 2.43, *p* = 0.048, *ηp*^2^ = 0.03^*^
**Oral health**	Attitudes towards sustainable dentistry	Pearson's correlation	*r*(344) = 0.11, *p* = 0.42^*^
	Willingness to compromise time and convenience		*r*(341) = 0.04, *p* = 0.421
	Willingness to pay more		*r*(343) = 0.09, *p* = 0.117
	Willingness to compromise the durability of dental treatments		*r*(343) = 0.03, *p* = 0.556
	Willingness to compromise appearance of teeth		*r*(343) = -0.08, *p* = 0.149
	Willingness to compromise health		*r*(343) = -0.04, *p* = 0.463
**Experiencing pain**	Attitudes towards sustainable dentistry	Independent t-test	*t*(342) = 0.45, *p* = 0.654, *d* = -0.08
	Willingness to compromise time and convenience		*t*(339) = 0.16, *p* = 0.870, *d* = -0.03
	Willingness to pay more		*t*(341) = -0.32, *p* = 0.750, *d* = 0.06
	Willingness to compromise the durability of dental treatments		*t*(341) = 0.13, *p* = 0.899, *d* = -0.02
	Willingness to compromise appearance of teeth		*t*(341) = 0.55, *p* = 0.586, *d* = -0.10
	Willingness to compromise health		*t*(40.26) = 1.33, *p* = 0.190, *d* = -0.28
Key: * = deno*t*es significan*t* resul*t*s

Appendix 1 List of questionnaire items not used in previous research **Attitudes towards sustainable dentistry (8 items):**

- I do not care about the environmental impact of dental services (r)
- I do not care if my dental treatments harm the environment (r)
- It is important to me that my dental treatments do not harm the environment
- It does not bother me whether or not my dental practice is environmentally friendly (r)
- It is important to me that my dental practice tries to reduce the impact on the environment
- The environmental impact of my dental work is not important to me (r)
- It is worthwhile trying to find a dental practice that is more environmentally friendly
- It is good to look after my teeth to reduce the impact of my dental work on the environment.

**Willingness to make compromises for sustainable dentistry **

- Time and convenience (7 items):
- I would be willing to wait longer for an appointment if my dental treatments were better for the environment
- I would be willing to change my dental practice to one that was more environmentally friendly
- I would be willing to visit the dentist with family if it was better for the environment
- I would be willing to visit a more environmentally friendly dentist even if it was more inconvenient to me
- I would be willing to have a longer appointment if my dental treatments were better for the environment
- I would be willing to visit the dentist less frequently if it was better for the environment
- I would not be willing to go out of my way to reduce the impact of my dental treatments on the environment (r).

**Money (3 items):**

- I would be willing to pay extra to reduce the impact of my dental treatments on the environment
- I would be willing to pay more if my dental treatments were better for the environment
- I would not be willing to pay more for environmentally friendly dental treatments (r).

**Durability (3 items):**

- I would be willing to have a filling that didn't last as long if it was better for the environment
- I would not be willing to have a less durable filling even if it was better for the environment (r).
- I would be willing to have a filling repaired, rather than replaced, if it was better for the environment.

**Aesthetics (5 items):**

- I would be willing to compromise the appearance of my teeth if it was better for the environment
- I would be willing to have a less than perfect smile if it was better for the environment
- I would be willing to have more noticeable dental work if it was better for the environment
- I would be willing to have a silver or gold, rather than a white, filling in my front tooth if it was better for the environment
- I would be willing to have a silver or gold, rather than a white, filling in my back tooth if it was better for the environment.

**Health (2 items):**

- I would be willing to compromise the health of my teeth for the environment
- I would not be willing to compromise the health of my teeth for the environment (r).
